# *KRAS*突变的非小细胞肺癌的研究进展

**DOI:** 10.3779/j.issn.1009-3419.2018.05.11

**Published:** 2018-05-20

**Authors:** 蕾 刘, 素菊 魏

**Affiliations:** 050011 石家庄，河北医科大学第四医院肿瘤内科 Department of Medical Oncology, Fourth Hospital of Heibei Medical Medical University, Shijiazhuang 050011, China

**Keywords:** KRAS, 肺癌, 预测, 预后, 靶向, KRAS, Lung neoplasms, Predictive, Prognostic, Target

## Abstract

肺癌是全球癌症相关死亡的主要原因。非小细胞肺癌（non-small cell lung cancer, NSCLC）占所有肺癌患者中的80%-85%，大多数肺癌患者在确诊时已处于晚期阶段。目前，基于驱动基因的靶向治疗的发展改变了晚期NSCLC患者的治疗模式。在NSCLC中，表皮生长因子受体突变（epidermal growth factor receptor, EGFR）和棘皮动物微管相关蛋白和间变性淋巴瘤激酶（echinoderm microtubule-associated protein-like 4-anaplastic lymphoma kinase, EML4-ALK）融合已被验证为强大的生物标志物。众所周知KRAS也是NSCLC中最常见的突变致癌基因之一，尽管20多年前在NSCLC中发现了*KRAS*突变，迄今为止用于治疗*KRAS*突变的NSCLC患者的药物有很多，但目前还没有针对直接消除KRAS活性的选择性和特异性抑制剂。此外具有*KRAS*突变的NSCLC患者对大多数系统性治疗的反应性差。然而使用靶向药物针对活化的信号通路个体化治疗对*KRAS*突变的NSCLC患者的预后有很好疗效。此外*KRAS*突变在NSCLC中的预后和预测作用尚不清楚。在这篇综述中，我们重点讨论了*KRAS*突变的NSCLC的研究进展，包括分子生物学、临床病理特征、*KRAS*突变的预后和预测等方面，进而有助于提高临床工作者对*KRAS*突变的NSCLC的认知。。

## Abstract (en)

Lung cancer is the leading cause of cancer-related deaths worldwide. Non-small cell lung cancer (NSCLC) accounts for 80%-85% of all patients with lung cancer, the majority of patients with lung cancer at the time of diagnosis is in the advanced stage. The development of target therapy based on has changed the mode of treatment in patients with advanced NSCLC. In NSCLC, epidermal growth factor receptor mutation (EGFR) fusion with echinoderm microtubule-associated protein-like4-anaplastic lymphoma kinase (EML4-ALK) has been shown to be a powerful biomarker. It is well known that KRAS is also NSCLC one of the most common mutations in oncogenes, although more than 20 years ago *KRAS* mutation was found in NSCLC. At present, although there are many drugs used to treat NSCLC patients with *KRAS* mutation, there is no selective or specific inhibitor for the direct elimination of KRAS activity. NSCLC patients with *KRAS* mutation have poor responsiveness to most systemic therapy. However, individualized therapy for activated signaling pathways with targeted drugs has a good effect on the prognosis of NSCLC patients with *KRAS* mutation. In addition, the prognostic and predictive role of *KRAS* mutation in NSCLC remains unclear. In this review, we focus on the research progress of NSCLC with *KRAS* mutation, including molecular biology, clinicopathological features, prognosis and prediction of *KRAS* mutation, which will help to improve the understanding of NSCLC in *KRAS* mutation.

肺癌是全球癌症相关死亡的主要原因。非小细胞肺癌（non-small cell lung cancer, NSCLC）占所有肺癌患者中的80%-85%，其中腺癌是NSCLC中最常见的病理类型，往往伴有致癌驱动突变如表皮生长因子受体突变（epidermal growth factor receptor, EGFR）和棘皮动物微管相关蛋白和间变性淋巴瘤激酶（echinoderm microtubule-associated protein-like 4-anaplastic lymphoma kinase, EML4-ALK）融合，并已研制出有效的酪氨酸激酶抑制剂和ALK抑制剂，改变了对NSCLC治疗策略。然而，15%-25%的NSCLC存在Kirsten大鼠肉瘤病毒基因同源物（*KRAS*）突变，其中白人比亚洲人群突变率更高（分别为25%-50%和5%-15%）^[1, 2]^。到目前为止KRAS致癌作用依然不明，并且没有一个有效针对这靶点的药物，进而*KRAS*突变的NSCLC患者的最佳治疗仍然是一个悬而未决的问题^[3]^。因此本文就*KRAS*突变的NSCLC的临床进展进行综述，有利于医护工作者提高对*KRAS*突变NSCLC的认知。

## 1 *KRAS*突变肺癌的分子及临床病理特征

KRAS在1984年NSCLC基因中首次被描述，是一种膜结合型的蛋白，定位于细胞膜内侧；同时位于EGFR信号通路上，对于肿瘤的发生及发展非常重要，正常情况下KRAS蛋白和CDP结合没有活性，当细胞外的生长分化因子把信号传到KRAS蛋白时，增强了其与GTP结合活性，使蛋白和GTP结合成为激活状态，信号系统开放。肿瘤细胞的生长、增殖、血管生成等过程都需要细胞内蛋白进行信号传导，而*KRAS*基因是传导蛋白的决定因素，*KRAS*突变型编码异常的蛋白，刺激促进恶性肿瘤细胞生长和扩散；并且不受上游EGFR的信号影响^[4-6]^。

众所周知*KRAS*突变在伴有吸烟史和组织类型是腺癌的年轻女性患者中突变率较高^[7]^。而*KRAS*突变包含颠换突变（嘌呤核苷酸替代嘧啶或反之亦然）或转移突变（嘌呤至嘌呤或嘧啶至嘧啶取代）两种类型。颠换突变在吸烟的患者中更为常见；而转移突变在不吸烟患者中更为常见^[4]^。绝大多数*KRAS*突变涉及外显子为12或13（G12C，G12D和G12V）置换突变，其中最常见G12C突变，其次是G12V突变。不同的基因突变可激活不同的信号通路，G12V和G12C点突变与增强的Ral下游信号传导相关；而G12D突变可激活磷酸肌醇3激酶（PI3K）途径^[8]^。在转移方面，*KRAS*突变型与KRAS野生型患者的转移部位或转移部位数量方面无差异，而一项纳入174例患者研究中表明*KRAS*突变是脑转移的独立危险因素（*P*=0.007）^[9]^。

此外，以前推测*KRAS*突变与*EGFR*突变和*EML4-ALK*易位是相互排斥的。然而最近数据显示伴*KRAS*突变的NSCLC可能是一个分子多样的实体，可以与*EGFR*突变或EML4-ALK易位同时存在^[10]^。Ulivi等^[10]^对380例患者进行*EGFR*及ALK基因检测，同时282例患者也进行*KRAS*突变分析，发现*KRAS*和*EGFR*以及*KRAS*和*EML4-ALK*双突变的患者分别占到1.1%和2.5%。其他分子基因如TP53/*STK11*突变与*KRAS*突变相关基因表达有明显相关性，*TP53*和*STK11*肿瘤抑制基因可能通过不同的机制促进肿瘤进展，其中*TP53*突变导致更大的增殖反应，*STK11*突变可抑制肿瘤免疫监视^[11]^。

## 2 *KRAS*突变作为NSCLC预后指标

*KRAS*突变状态作为预后标志物仍然存在争议。多数研究显示*KRAS*突变是NSCLC中的阴性预后指标。一项荟萃分析显示*KRAS*突变NSCLC存活率较低（HR=0.35; 95%CI: 16-1.56）^[7]^。目前认为*KRAS*突变作为预后标志物取决两个方面，其一，为*KRAS*基因不同的突变状态。在一项包括677例患者的研究中，Yu等^[12]^证实G12C/G12V突变肿瘤患者的中位总生存期与其他所有点突变患者的差异无统计学意义，两者均为1.2年（P=0.74）。与*KRAS*密码子12突变的患者相比，密码子13突变患者的死亡风险增加（HR=1.50; 95%CI: 1.11-2.04; *P*=0.009）。在控制年龄，性别和吸烟史的多因素分析中，KRAS密码13突变的总体生存期较KRAS密码子12突变短。其二，肿瘤的分期也是KRAS作为预后标记物的影响因素。对于可切除的肿瘤，KRAS的预后价值似乎很小。在早期切除的NSCLC四项辅助化疗中的预后和预测效果分析数据中显示^[7]^，不同*KRAS*突变状态下300例患者的总生存期无统计学差异。值得注意的是，在少数伴密码子13突变患者中，辅助化疗使其总体生存期缩短。此外Tomasini等^[13]^发现，局部治疗后KRAS患者相对于EGFR突变型和野生型肿瘤的脑转移复发率较低。

## 3 KRAS突变对NSCLC相关治疗药物的预测意义

### 3.1 *KRAS*突变对免疫检查点抑制剂疗效的预测意义

最近Chen等^[14]^的研究纳入216例NSCLC患者，69例为EGFR/ALK野生型肺腺癌患者，其中19例患者携带*KRAS*突变。通过倾向匹配评分分析后与50例EGFR/ALK/KRAS野生型患者中38例患者的基线特征相匹配。最后，通过免疫组织化学检测匹配后的57例患者组织中的细胞程式死亡-配体1（programmed cell death 1 ligand 1, PD-L1）表达。表明肺腺癌伴*KRAS*突变患者PDL-1表达明显高于KRAS野生型，并且发现*KRAS* G12D突变激活了磷酸化细胞外信号调节激酶1/2（phosphorylated extracellular regulated kinase 1/2, p-ERK1/2）和磷酸化AKT（p-AKT），应用ERK1/2抑制剂（SCH772984）抑制p-ERK，进一步导致PD-L1表达的降低。表明PD-L1表达受KRAS G12D突变的调控。此外Dong等^[15]^研究支持TP53和*KRAS*突变可作为指导抗PD-1/PD-L1免疫治疗的一对潜在预测因素。Scheel等^[16]^发现具有突变型*KRAS*，突变型TP53和野生型STK11的标本中PD-L1阳性表达最高。相反，具有野生型KRAS，野生型TP53和突变型STK11的标本PD-L1阳性表达最低。

### 3.2 *KRAS*突变对EGFR抑制剂疗效的预测意义

由于KRAS位于EGFR受体的下游，因此*KRAS*突变会使EGFR酪氨酸激酶抑制剂（TKI）的治疗效果降低。12项前瞻研究表明*KRAS*突变可以作为EGFR-TKIs治疗反应的不良预测分子标志物，此外的证据表明KRAS状态位点的特异性可能导致对EGFR-TKI的敏感性差异^[17]^。Zer等^[18]^在接受EGFR-TKI治疗的275例*KRAS*突变患者的临床研究中证实鸟嘌呤-胸腺嘧啶核苷转换突变的患者比具有鸟嘌呤-腺嘌呤转换突变的OS更长（*P*=0.01）。其中*G12C*/*G12V KRAS*突变患者经EGFR-TKI的治疗后预后差，而*G12D*/*G12S KRAS*突变患者可以从EGFR-TKI中获益，但由于数量少，约占总*KRAS*突变的50%，观察结果需要进一步证实。

### 3.3 *KRAS*突变对临床化疗疗效的预测意义

常规的细胞毒性化疗仍然推荐用于*KRAS*突变NSCLC患者，但*KRAS*突变作为化疗预测标志存在很大争议。最近相关研究表明不同的*KRAS*突变类型在化疗中的敏感性不同。Park等^[19]^观察到*KRAS* G12C突变与顺铂的反应降低和培美曲塞的反应增加密切相关，然而G12V突变对顺铂的敏感较高，对培美曲塞的反应略低。相关研究显示在G12V患者中，观察到紫杉烷类药物的ORR明显改善（*P* < 0.01），表示G12V患者对紫杉烷治疗反应较好^[20]^。总之，不同*KRAS*突变类型可能影响对化疗的反应性。

## 4 抑制KRAS下游通路的靶向治疗的临床进展

由于*KRAS*突变在NSCLC中的较高的表达，越来越引起专家及学者的重视。尽管肺癌靶向治疗取得了飞速发展。但是，直接针对KRAS靶点抑制剂的药物研发受到KRAS生物化学复杂性的挑战，未能取得良好疗效。如上述KRAS下游的信号传导是肺肿瘤发生的根本驱动因素，因此抑制KRAS下游通路可能是种有效的治疗策略，其抑制途径包括RAF-MEK-ERK和PI3K-AKT-mTOR途径的抑制。

### 4.1 抑制焦点粘附激酶（FAK）

FAK是KRAS信号传导的下游效应因子。临床前数据显示，在突变*KRAS* NSCLC细胞中，FAK抑制导致持续的DNA损伤^[21]^。FAK抑制剂defactinib的Ⅱ期针对实体瘤的试验中，晚期预处理的KRAS阳性NSCLC患者根据生物标志物如p16和p53分为5组。用defactinib治疗耐受良好，呼吸困难和恶心是最常见的3级不良事件（各为7.3%），PFS为11.7周，DCR为61%^[22]^。

### 4.2 MEK抑制剂

MEK（丝裂原活化蛋白激酶）是MAPK信号级联的下游效应物。由于KRAS不易于定位，所以最初认为MEK可能是一个合适的目标。然而，MEK抑制剂作为单一疗法在临床试验中的疗效局限^[23]^。Selumetinib（AZD6244）是一种有效，高选择性的MEK抑制剂。临床前研究结果表明，Selumetinib在体内显著抑制了*KRAS*突变NSCLC异种移植瘤的肿瘤生长。Ⅱ期临床试验结果显示与多西紫杉醇联合能提高NSCLC患者的中位PFS（5.3个月*vs* 2.1个月）^[24]^。但名为SELECT-1的双盲、随机Ⅲ期临床试验中数据说明selumetinib联合多西他赛较单独应用多西他赛并不能改善携带*KRAS*突变的NSCLC患者的总体生存期和无进展生存期，并且副作用较为显著，因此目前迫切需要开发其他新药来治疗这一类NSCLC^[25]^。

### 4.3 PI3K通路抑制剂

PI3K是KRAS下游的效应激酶，并且是PI3K/AKT/mTOR途径的一部分，是多个途径融合的场所，因此对其调节是复杂的。大约2%的NSCLC发现*PI3KCA*突变，并且独立于KRAS激活导致细胞增殖和存活。研究显示使用BKM120抑制EGFR和KRAS下游PI3K途径从而抑制*KRAS*突变的NSCLC细胞系的生长^[26]^。

### 4.4 mTOR抑制剂

mTOR是PI3K/AKT/mTOR途径中PI3K下游的丝氨酸/苏氨酸激酶。临床前数据表明，mTOR抑制剂阻断*KRAS*突变型肺腺癌小鼠模型中的肿瘤生长。已经在NSCLC治疗中研究了几种mTOR抑制剂（例如依维莫司，阿糖胞苷）。Ridaforolimus（Deforolimus/MK-8669）是一种新的雷帕霉素类似物, 是一种新型有效的mTOR选择性抑制剂。在Ⅱ期临床试验中，70例*KRAS*突变NSCLC患者接受了8周的Ridaforolimus。8周后，28例被认为稳定性疾病的患者被随机分配接受安慰剂或继续Ridaforolimus，与对照组相比，Ridaforolimus的PFS显着升高（4个月*vs* 2个月；HR=0.36；*P*=0.133），OS更好（18个月*vs* 5个月；HR=0.46；*P*=0.09）^[27]^。因此，mTOR是治疗*KRAS*突变NSCLC的有希望的靶标。

## 5 KRAS突变NSCLC患者的其他靶向治疗进展

### 5.1 阿帕替尼

血管内皮生长因子（vascular endothelial growth factor, VEGF）信号通过激活VEGF受体（VEGF receptor, VEGFR）促进血管生成，VEGF受体是癌症治疗的关键目标。在晚期肺腺癌，尤其是*KRAS*突变患者中，抗血管生成药物单一疗法作为二线治疗后的证据很少。阿帕替尼是VEGFR-2酪氨酸激酶的口服小分子抑制剂。以前的报道已经证明了其在晚期乳腺癌和胃癌治疗中的效率，在Zeng等^[28]^研究中共纳入4例患者，所有患者均为*KRAS*突变的年龄在56岁-81岁之间男性Ⅳ期腺癌。在口服阿帕替尼治疗（250 mg/d）之前，所有患者接受一线和二线化疗。阿帕替尼单药治疗1个月后，3例患者出现SD，1例患者出现PD。3例患者的无进展生存期（progression-free survival, PFS）分别为1.5个月、4.5个月和5.5个月。只有1例患者表现出可控的声音嘶哑和咯血（1级）。因此在晚期*KRAS*突变的腺癌患者中，阿帕替尼或许可作为挽救治疗的选择。但是，在*KRAS*突变的NSCLC患者中进行关于阿帕替尼的大规模随机对照临床试验来验证。

### 5.2 CDK4/6抑制剂

在*KRAS*突变的肺腺癌模型中，发现KRAS和CDK4共表达促进视网膜母细胞瘤蛋白（Rb）磷酸化，最终导致人类肿瘤发生。因此，抑制CDK4可能是*KRAS*突变阳性NSCLC治疗中最有吸引力的靶点，其中Abemaciclib（LY2835219）是一种选择性CDK4/6抑制剂，有效抑制细胞培养中癌细胞的生长，在*KRAS*突变NSCLC的模型中显示出治疗效力^[29]^。此外Tao等^[30]^研究显示palbociclib与trametinib的组合被证明在*KRAS*突变的NSCLC患者中具有显著疗效。目前正在具有*KRAS*突变的NSCLC患者中进行随机Ⅲ期临床试验，比较abemaciclib（一种有效的CDK4/6抑制剂）联合最佳支持治疗（BSC）与厄洛替尼联合BSC的疗效。

### 5.3 多激酶抑制剂

在治疗*KRAS*突变的NSCLC研究最多的是索拉非尼，一种多激酶抑制剂。其抑制VEGFR，血小板衍生生长因子受体（platelet-derived growth factor receptor, PDGFR），B-RAF的丝氨酸/苏氨酸激酶活性和RAF-1，并被批准用于肾细胞癌，肝细胞癌和分化型甲状腺癌。索拉非尼具有双重的抗肿瘤作用，通过作用于VEGFR抑制新生血管的形成，通过阻断由RAF/MEK/ERK介导的细胞信号传导通路而直接抑制肿瘤细胞的增殖，在Li等^[31]^研究中表明索拉非尼可显著抑制*KRAS*突变的NSCLC细胞的生长。此外，索拉非尼与培美曲塞联合治疗*KRAS*突变的NSCLC时具有显著协同作用。

## 6 *KRAS*突变NSCLC患者的免疫治疗

使用PD-1/PD-L1抑制剂能够恢复抗肿瘤的免疫力，并且已经在广泛的肿瘤类型（鳞状、腺癌）中表现出功效。涉及3, 025例患者的荟萃分析显示免疫检查抑制剂延长*KRAS*突变亚组的总生存期（HR=0.65; 95%CI: 0.44-0.97; *P*=0.03）^[32]^。而在Diwakar Davar的报告中，描述了在伴*KRAS*突变综合治疗后的晚期肺腺癌患者，单剂量nivolumab治疗后复查发现所有病变均明显缩小（接近完全缓解），2个非靶病变（左胸膜结节和食管旁淋巴结）完全消失^[33]^。有理由相信免疫检验点抑制剂将来成为征服*KRAS*突变NSCLC的希望。

## 7 治疗前景

目前新型药物如TKI和免疫检查点抑制剂已经迅速改变了NSCLC的治疗策略并改善了患者预后。但针对*KRAS*突变的临床治疗方式仍面临困难，目前临床研究显示组合方法是可行的。并且本综述所示，多项证据表明，*KRAS*基因的不同基因突变显示出不同的预测意义，今后需要针对不同的患者使用的高度特异性靶向药物才能实现疗效的最大化。目前靶向治疗和常规治疗相联合是一个具有前景的治疗方式。总之，我们期待着不久的将来，*KRAS*突变的NSCLC的临床治疗不再是难题。
